# The Usefulness of Virtual Reality in Symptom Management during Chemotherapy in Lung Cancer Patients: A Quasi-Experimental Study

**DOI:** 10.3390/jcm13154374

**Published:** 2024-07-26

**Authors:** Lucia Mitello, Flavio Marti, Lucia Mauro, Ludovica Siano, Antonello Pucci, Concetta Tarantino, Gennaro Rocco, Alessandro Stievano, Laura Iacorossi, Giuliano Anastasi, Rosaria Ferrara, Anna Rita Marucci, Giustino Varrassi, Diana Giannarelli, Roberto Latina

**Affiliations:** 1 Department of Health Professions, San Camillo-Forlanini Hospital, 00152 Rome, Italy; lmitello@scamilloforlanini.rm.it (L.M.); lmauro@scamilloforlanini.rm.it (L.M.); antonello.pucci@scamilloforlanini.rm.it (A.P.); ctarantino@scamilloforlanini.rm.it (C.T.); ar.marucci@gmail.com (A.R.M.); 2 School of Nursing and Midwifery, Faculty of Medicine and Psychology, Sapienza University of Rome, 00152 Rome, Italy; 3 Emergency Department, Fatebenefratelli Hospital, 00189 Rome, Italy; ludovica.siano@libero.it; 4 Center of Excellence for Nursing Scholarship, OPI of Rome, 00136 Rome, Italy; genna.rocco@gmail.com; 5 Faculty of Medicine, Catholic University Our Lady of Good Counsel, 1005 Tirana, Albania; 6 Department of Clinical and Experimental Medicine, University of Messina, 98122 Messina, Italy; alessandro.stievano@unime.it; 7 Department of Life, Health and Health Professions Sciences Link Campus University, 00165 Rome, Italy; l.iacorossi@unilink.it; 8 Department of Trauma, AOU G. Martino University Hospital, 98124 Messina, Italy; giuliano.anastasi@polime.it; 9 Department of Anatomy Histology, Legal Medicine and Orthopaedics, Sapienza University of Rome, 00161 Rome, Italy; rosaria.ferrara@uniroma1.it; 10 Research Department, Paolo Procacci Foundation, 00193 Rome, Italy; giuvarr@gmail.com; 11 Facility of Epidemiology and Biostatistics, IRCCS Policlinico Gemelli, 00168 Rome, Italy; diana.giannarelli@policlinicogemelli.it; 12 Department of Health Promotion Science, Maternal and Infant Care, Internal Medicine and Medical Specialties, University of Palermo, 90127 Palermo, Italy; roberto.latina@unipa.it

**Keywords:** virtual reality, lung cancer, pain perception, chemotherapy, well-being, nursing

## Abstract

**Background:** Virtual reality (VR) emerges as a promising non-pharmacological intervention for managing symptoms and providing distraction during chemotherapy. This study aims to assess VR’s effectiveness on cancer-related symptoms, vital signs, and the patients’ perception of chemotherapy in lung cancer patients. **Methods**: A quasi-experimental study was conducted on 100 patients. Participants were allocated into an intervention group (n = 55), which experienced immersive VR, and a comparison group (n = 45), which received usual care. Data were collected through questionnaires and checklists, including feedback on the VR experience, pain, vital signs, and common cancer symptoms, assessed through the Edmonton Symptom Assessment Scale. **Results**: VR had a significant impact on reducing the perception of chemotherapy length. Patients reported high levels of satisfaction and tolerability. No adverse events were observed. VR did not have significant influence on pain intensity or vital signs. The only exceptions were oxygen saturation, where a significant difference (*p* = 0.02) was reported, and the perception of chemotherapy duration. **Conclusions**: As a non-pharmacological intervention, VR proves to be beneficial in minimizing the perceived length of chemotherapy sessions for lung cancer patients, enhancing their overall treatment experience. The intervention was found to be a safe, feasible, and well-accepted distraction technique. Future research should explore VR’s potential effects on a wider range of symptoms and evaluate its impact on long-term outcomes.

## 1. Introduction

Cancer represents a critical challenge to global public health, as evidenced by the World Health Organization (WHO), which identifies it as a leading cause of mortality before the age of 70 worldwide [1]. Recent data for 2020 reveal that Europe has high cancer incidence and mortality rates, at 22.8% and 19.6%, respectively, second only to Asia [2]. Among the different types of cancer, breast cancer emerges as the most prevalent globally (11.7%), closely followed by lung cancer (11.4%), which holds the highest mortality rate (18%), with 2.21 million new cases and 1.8 million deaths reported in 2020 [3]. The treatment landscape for lung cancer is varied and tailored to the disease’s stage and type, including surgery, chemotherapy, radiotherapy, immunotherapy, and palliative care options [4,5]. Over the years, chemotherapy has emerged as the cornerstone of lung cancer therapy [6], currently representing the primary modality of treatment [7], especially for advanced stages of the disease [8]. However, chemotherapy is associated with a spectrum of side effects [9], ranging from physical symptoms, such as fatigue, pain, and nausea, to psychological consequences, such as anxiety and depression, impacting lung cancer patients’ quality of life [10,11,12,13,14]. The mentioned side effects can increase the treatment burden and negatively influence adherence to chemotherapy protocols [15,16,17], which is further affected by socio-economic and living conditions [18,19]. Studies show that nearly 29% of lung cancer patients might receive chemotherapy differently than recommendations [20], and up to 12% may not comply with the prescribed treatment procedures [21]. In the oncological setting, non-adherence not only implies significant economic costs to healthcare systems [22] but can also lead to worsened clinical outcomes [23], adversely affecting lung cancer patients’ prognoses [24]. Therefore, developing and implementing strategies to enhance chemotherapy adherence in lung cancer patients represent a priority in oncology nursing [25,26,27].

The scientific literature increasingly emphasizes the potential of non-pharmacological interventions to improve the well-being of lung cancer patients undergoing chemotherapy. This interest is evidenced by several studies exploring strategies such as acupressure [28,29], physical exercise [30], relaxation techniques [31], yoga [32,33], music therapy [34], and meditation [35]. The innovative use of virtual reality (VR) during chemotherapy sessions has recently been proposed as a novel non-pharmacological intervention to enhance patient well-being, showing the evolving panorama of supportive cancer care [36]. VR represents a rapidly advancing technology characterized by many definitions that reflect its complexity and multifaceted nature [37]. In contemporary healthcare, VR is “a three-dimensional computer-generated simulated environment, which attempts to replicate real world or imaginary environments and interactions, thereby supporting work, education, recreation, and health” [38]. VR is classified into two main categories: non-immersive and immersive [39]. Non-immersive VR employs multiple screens to simulate environments around the user. Immersive VR uses head-mounted displays (HMDs) to achieve total sensory immersion in a virtual environment, enhancing the user’s experience [40]. VR has been effectively utilized across various populations to enhance well-being, including patients with dementia [41], healthcare workers [42], and the general population during the COVID-19 pandemic [43]. In the medical settings, VR has shown significant efficacy in reducing patients’ fear, pain, and distress related to medical procedures [44,45], as well as in mitigating symptoms of anxiety, depression, and fatigue [46]. Its application in oncology, specifically during chemotherapy, has gained recognition for its capability to offer distraction [47], thereby reducing anxiety, depression, fatigue, heart rate, and blood pressure in adults while decreasing symptoms such as anxiety, nausea, and pain among pediatric patients [36,48,49,50]. Furthermore, VR interventions have been observed to decrease anxiety, depression, fatigue, and the perceived duration of chemotherapy sessions in breast and ovarian cancer populations [51,52,53,54], and to improve quality of life and reduce anxiety in leukemia patients [55]. However, the existing research on this topic is characterized by its variable quality and the need for more homogeneity [56,57]. Moreover, there are limited and dated studies specifically focused on investigating the utilization of VR during chemotherapy in lung cancer patients, though the findings are encouraging [58,59].

Considering the existing literature and the efficacy of distraction as a non-pharmacological intervention that does not require specialized training for nursing staff and has no side effects [60,61], this study aims to investigate the impact of immersive VR on the well-being, vital signs, and chemotherapy experience of lung cancer patients. We hypothesize that immersive VR has the potential to significantly alleviate common symptoms associated with cancer and enhance the overall well-being and the experience of chemotherapy for lung cancer patients, with minimal to no adverse effects.

## 2. Aims

This study aimed to assess the effectiveness of immersive VR distraction technology in managing side effects among lung cancer patients during chemotherapy. The primary endpoint of this study was to compare the outcome in terms of the Edmonton Symptom Assessment Scale (pain, tiredness, nausea, depression, anxiety, drowsiness, appetite, well-being, and shortness of breath) and vital parameters between patients assigned to the VR arm and those of the control group. Secondary endpoints were chemotherapy duration perception, adherence, and safety.

## 3. Methods

### 3.1. Study Design

We adopted a quasi-experimental study design, incorporating an intervention and a comparison group. Participants allocated to the intervention group experienced immersive VR during their first chemotherapy session, while those in the comparison group received usual care. The study’s design and reporting were guided by the principles of the Transparent Reporting of Evaluations with Non-randomized Designs (TREND) Statement Checklist [62] to ensure the clarity and replicability of our methods (see Supplementary Materials).

### 3.2. Participants and Setting

The study was conducted in the Pneumological Oncology Unit of a healthcare facility in A.O. San Camillo-Forlanini Hospital in Rome, Italy. Participants were eligible if they were 18 years or older, of both sexes, diagnosed with any stage of lung cancer, Easter Cooperative Oncology Group (ECOG) Performance Status ≤ 2 (ambulatory and capable of all selfcare but unable to carry out any work activities; up and about more than 50% of waking hours) [63], scheduled to undergo their first chemotherapy session, proficient in the Italian language, willing to participate, and able to provide informed consent. Exclusion criteria included people undergoing chemotherapy for palliative purposes, a diagnosis of any neurological, psychiatric, or cognitive disorders, the current use of analgesic, antipsychotic, sedative drugs, or psychoactive substances, ECOG Performance Status > 2, and having visual or hearing impairments that might influence the VR experience. Recruitment was based on a non-probabilistic consecutive sampling method, assigning individuals to the intervention group if they visited the oncology unit on even-numbered days and to the control group if they arrived on odd-numbered days. The recruitment process continued until the target sample size of 100 participants was reached, ultimately comprising 55 patients in the intervention group and 45 in the control group. Within the intervention group, one participant declined to have his vital signs monitored before the intervention, and two still needed to complete the post-intervention assessments. Consequently, 53 patients from the intervention group and 45 from the comparison group were considered in the post-intervention analysis.

### 3.3. Intervention

The intervention was a single session of immersive VR coinciding with the duration of scheduled chemotherapy treatment for participants in the intervention group. The control group received usual care, characterized by the standard nursing support provided during chemotherapy sessions. The study used five VR devices, each comprising a head-mounted display (HMD) for immersive visual content, a bone conduction headset to deliver audio, and a remote control for user-guided exploration and navigation within the virtual environment. The HMDs were designed for comfort and adjustability to ensure a personalized fit, optimizing the visual experience for each participant. Before initiating the VR session, oncology nursing staff, trained specifically for this study, equipped participants with the HMDs, explaining the use and adjustment procedures to maximize comfort and immersion. To maintain strict hygiene standards, each HMD was paired with disposable face masks and caps to cover participants’ faces and heads, while remote controls were maintained in disposable plastic covers. Following the VR intervention, the equipment underwent thorough cleaning and sterilization in line with the hospital’s infection control protocols, ensuring safety and hygiene for each use. Participants in the VR group were offered a selection of five virtual scenarios: rivers and waterfalls, lakes, rivers and forests, mountains, and Niagara Falls. Accompanying these visuals, the HMDs provided ambient sounds to complement the visual scenery, with volume control and sound muting options available via the remote control. This feature allowed participants to adapt their auditory experience to their comfort level. The remote control also enabled users to navigate the different virtual scenarios, enabling participants to customize their experience and interact with the virtual environments during their chemotherapy treatment. The nursing staff remained available throughout the intervention to offer further instructions, answer any questions, and address potential adverse effects.

### 3.4. Outcome Measurements

A comprehensive suite of tools, including questionnaires, scales, and checklists, was utilized to evaluate the impact of the immersive VR intervention on the study’s variables. Detailed documentation of these tools, including the questionnaire for participants and the checklist used by nursing staff, is available in the Supplementary Materials.

#### 3.4.1. Socio-Demographic Information

A structured self-reported questionnaire was used to collect socio-demographic data from participants. This included sex, age, geographic provenience, marital status, living situation, education level, and employment status.

#### 3.4.2. Primary Outcomes

##### Edmonton Symptom Assessment Scale (ESAS)

The ESAS is a valid and reliable self-report instrument for evaluating symptom burden among cancer patients [64]. It comprises nine items on a numerical rating scale (NRS) ranging from 0 (no symptom) to 10 (worst possible symptom), allowing patients to self-report the severity of symptoms such as pain, tiredness, nausea, depression, anxiety, drowsiness, appetite, well-being, and shortness of breath. Scores for each symptom are recorded individually, and a total symptom burden score is calculated as the sum of all item scores. The instrument is validated in Italian, and the translated version demonstrated strong reliability and validity [65].

##### Vital Signs

An objective assessment of the patient’s physical health status was performed through multi-parameter monitoring equipment, capturing systolic and diastolic blood pressure, heart rate, respiratory rate, body temperature, and oxygen saturation.

#### 3.4.3. Secondary Outcomes

##### Patient-Reported Data on VR Intervention

An ad hoc self-report questionnaire was designed to collect feedback from the intervention group on their experience with the VR intervention. It covered aspects such as virtual scenario(s) experienced, satisfaction with the chosen scenario(s), the use of audio support, any interruptions and their causes, comfort with the VR equipment, and perceived chemotherapy session duration. Control group participants also provided estimates of their chemotherapy session length via a single-item questionnaire to facilitate comparative analysis. The oncology nursing staff employed a structured checklist to document the safety and logistical aspects of the VR intervention, including the start and end times of chemotherapy sessions, vital signs recorded, and any adverse events noted during the VR intervention.

### 3.5. Data Collection

The data were collected from April to December 2021. Data collection occurred at two time points: before the start of the chemotherapy session (T0) and after the chemotherapy session (T1). At T0, socio-demographic characteristics, vital signs, and ESAS scores were collected from intervention and control group participants. At T1, these measurements were repeated, excluding the socio-demographic data, and participants in the intervention group also completed the questionnaire designed to capture their VR experience. Nursing staff recorded the duration of the chemotherapy session, any adverse events, and pre- and post-chemotherapy vital signs using the structured checklist. All participants were assigned a unique identifier code used across questionnaires and checklists to ensure privacy and confidentiality.

### 3.6. Data Analysis

The analysis was conducted using descriptive statistical methods. Categorical variables were summarized using frequencies and percentages, while continuous variables were described with mean values, standard deviations (SD), and weighted means (WM). The Chi-square test was employed to explore associations between variables, and the independent Student’s *t*-test was used to compare the intervention and control groups. The Kolmogorov–Smirnov test assessed the normality of the data distribution. A significance threshold was set at *p* < 0.05 for all tests. Data analysis was performed using SPSS (Statistical Package for the Social Sciences) for Windows, version 20.0 (IBM Corp. Armonk, NY, USA).

### 3.7. Ethical Considerations

The research received approval from the independent Ethics Committee Lazio 1 (protocol number 1102-2018/EC), and institutional consent was secured from the hospital. Eligible participants were informed about the study’s purpose and their right to withdraw at any time without any consequences. Written informed consent was obtained from all participants prior to their inclusion in the study, ensuring voluntary participation. The research adhered to the ethical standards outlined in the Declaration of Helsinki and the Good Clinical Practice Guidelines, ensuring that the participants’ rights, safety, and well-being were protected throughout all the study’s phases.

## 4. Results

### 4.1. Participants Characteristics

Table 1 provides a comprehensive overview of the socio-demographic characteristics of the participants involved in this study. Our sample consisted of subjects suffering from Non-Small-Cell Lung Cancer (NSCLC) (82.0%), Small-Cell Lung Cancer (SCLC) (16.0%), and Malignant Pleural Mesothelioma (MPM) (2.0%). In the VR group, 50.9% were male. In the control group, 49.1% were male. The mean age of the VR patients was 67.4 (DS = 7.3), and their BMI was 27 (DS = 4.3) versus CTRL 27.1 (DS = 4.9). All samples comprised 48% females, 67% were married, 56% had high school diplomas and university degrees, 84% lived with others, 67% were retired, and 66% had no pain. The mean age was comparable between the intervention group (67.4 ± 7.3 years) and the comparison group (67.2 ± 8.5 years), with a non-statistically significant difference observed (*p* = 0.058). Likewise, no significant differences were identified in geographic provenience, marital status, living situation, education level, and employment status between the two groups. Initial assessments of symptom burden and vital signs showed no significant differences between the intervention and comparison groups at baseline.

### 4.2. Primary Outcomes

#### 4.2.1. Impact of Immersive VR on the Edmonton Symptom Assessment Scale

As far as the results of the indicators (self-reported severity of symptoms such as pain, tiredness, nausea, depression, anxiety, drowsiness, appetite, well-being, and shortness of breath) identified by the ESAS results are concerned, the use of virtual reality does not seem to have a statistically significant impact. The analysis of ESAS immediately before and after each VR session seems to show no significant reduction in pain, depression, anxiety, shortness of breath, or improved well-being. VR positively affects the sense of appetite (*p* = 0.08) (Table 2).

#### 4.2.2. Effects of VR on Vital Signs

The evaluation of the immersive VR intervention’s effect on primary outcomes revealed no significant differences in the overall burden of common cancer symptoms or vital signs between the intervention and comparison groups, as reported in Table 3. The only exception was oxygen saturation, significantly better in the experimental group (*p* = 0.02). The equivalence in baseline measures provides a robust foundation for evaluating the effects of the immersive VR intervention on the study outcomes.

### 4.3. Secondary Outcomes: Feasibility, Adherence, Perceived Chemotherapy Duration, and Safety

The analysis focusing on the immersive VR intervention group highlighted positive outcomes regarding feasibility and adherence. Most participants (50 = 94.3%) engaged with more than one virtual scenario offered, and 34 (64.1%) explored all five scenarios, indicating a high level of commitment to the VR intervention. Participant satisfaction with each virtual environment was high, with all scenarios receiving an average score above 5 on a 7-point Likert scale, indicating good satisfaction levels. The ‘Lakes’ scenario emerged as the favorite, with a WM satisfaction score of 5.9, closely followed by ‘Rivers and Forests’ (WM = 5.8), ‘Niagara Falls’ (WM = 5.7), ‘Rivers and Waterfalls’ (WM = 5.6), and ‘Mountains’ (WM = 5.2). Audio support enhanced the VR experience for over half of the intervention group (28 = 52.8%).

Regarding tolerance, 60.4% (n = 32) of participants reported experiencing good comfort with the VR equipment, while 39.6% (n = 21) reported less favorable acceptance. Moreover, 8 patients (15%) opted to discontinue the VR experience prematurely, claiming for discomfort (n = 6) and boredom (n = 2) as their primary reasons.

A significant finding was the difference in the perceived duration of chemotherapy sessions between the intervention and comparison groups. The intervention group reported a perceived duration significantly shorter than the actual time (real duration = 69.06 ± 44.75 min; perceived duration = 48.72 ± 40.11 min; *p* < 0.001). In contrast, the comparison group perceived a duration closely matching the actual length (real duration = 73.70 ± 48.05 min; perceived duration = 68.18 ± 46.39 min; *p* < 0.29). These data underscored the potential of VR to positively influence the perception of time during chemotherapy. Notably, the nursing staff did not observe any adverse events related to the VR treatment, further affirming the safety of the immersive VR intervention within the studied population.

## 5. Discussion

This quasi-experimental study explored the impact of immersive VR on symptom management and the effects of the chemotherapy experience in lung cancer patients. Contrary to our initial hypothesis and other studies [66,67], demonstrating that a one-time VR intervention is sufficient to reduce pain significantly, tiredness, drowsiness, shortness of breath, depression, and anxiety measured by ESAS in a group of terminal cancer patients, we did not find such effects. Our results analysis of ESAS immediately before and after each VR session showed no significant reduction in pain, depression, anxiety, shortness of breath, and improved well-being in the overall burden of common cancer symptoms or vital signs between the intervention group and the comparison group. The evidence on the clinical effectiveness of VR is limited. One recent review described that qualitative and quantitative data on patient outcomes are limited and originate from studies conducted in single geographical locations with small sample sizes [68]. Moreover, diverse assessment measures were employed to measure the outcomes of VR interventions, which were responsible for difficulties in comparison. The only exception was represented by oxygen saturation, with a significant difference between our two groups. The distraction achieved by VR could provide a person with greater relaxation and greater control of breathing in a context of immersion with a virtual (but realistic) nature, different from the hospital context where patients were undergoing chemotherapy. This could explain the improvement in saturation level. Moreover, using VR, patients can imagine being in motion, in the open air, and this may have contributed. Perhaps this topic deserves further investigation, assuming that oxygen saturation is a key parameter in chemotherapy.

However, the study uncovered significant findings related to patient engagement and satisfaction with the VR intervention and a significant change in patients’ perception of the duration of chemotherapy sessions. The lack of significant improvements in common cancer symptoms among participants may be attributed to the distinct symptom profile associated with lung cancer, which is often characterized by more severe and complex symptomatology compared to other cancer types [16,69,70]. Furthermore, the demands of chemotherapy treatments may further complicate symptom management [71], exacerbating issues such as dyspnea, fatigue, pain, and reduced quality of life [72,73,74,75,76]. Therefore, the intense symptom burden inherent to lung cancer, alongside the complex impact of chemotherapy, may limit the perceived effectiveness of VR as a non-pharmacological intervention for symptom management within this population, despite VR’s success in other adult and pediatric cancer cohorts [44,77,78].

Concerning vital signs, our results are partially similar to previous findings in oncology. Studies by Ioannou [46] and Menekli [79] have reported minimal to moderate changes in vital signs following VR interventions in adult and pediatric cancer patients, respectively. The variance in our findings may reflect the specific physiological and psychological states of lung cancer patients undergoing chemotherapy, suggesting that VR alone could not induce significant alterations in vital signs in this group. This emphasizes the role of VR as a potential and effective distractive strategy rather than a direct influencer of physiological parameters.

About pain management, the clinical trial of Bani Mohammad et al. [80] showed that VR technology significantly reduced patients’ pain. Their data are in agreement with other researchers who used VR distraction interventions during painful procedures [81]. Moreover, a recent review investigated VR for pain management: only two studies reached statistical significance, but the power of their results was diminished because of the small sample sizes of fewer than 20 patients in either study [82]. There are other data showing that VR can be an effective [44] and safe adjuvant pain therapy. However, several issues must be addressed before VR is widely accepted as a routine intervention in pain conditions [83]. Pediatric cancer patients in the intervention group with VR demonstrated a more significant reduction in pain (estimated mean difference = −1.69, *p* = 0.007) and anxiety levels (estimated mean difference = −3.50, *p* < 0.001) compared with the control group [78]. According to our results, the effectiveness of immersive VR in reducing pain was unclear. Distraction analgesia is the most well-known mechanism attributed to the impact of VR on pain. However, a modest scientific production supports its efficacy, and further robust assessment of effectiveness is required before any clinical recommendations can be made [61,83,84].

Our feasibility, adherence, and safety findings indicate that immersive VR represents a promising, well-tolerated, non-pharmacological approach that can significantly improve the chemotherapy experience in lung cancer patients, significantly reducing time perception. The VR intervention seems to be appreciated by participants, and no one reported adverse side effects caused by its use. This aligns with the literature highlighting VR’s efficacy in modifying time perception within virtual environments [85,86] and its safe application as a distraction strategy for cancer patients during chemotherapy [47,80,87]. Furthermore, our results mirror prior studies indicating VR’s capability to reduce perceived chemotherapy duration among cancer patients, including those with lung cancer [58,59].

### Limitations

Despite its contributions, this study has limitations. The quasi-experimental design, non-randomized sampling, the absence of data regarding the correlation between types of chemotherapy and their side effects, and the relatively small sample size may introduce biases, potentially affecting the results’ generalizability. Additionally, the investigation focused on a single VR session, leaving the long-term effects of continued VR use on patient outcomes and treatment adherence to be explored.

## 6. Implications for Clinical Practice

The high level of engagement and satisfaction with the VR intervention underscores its potential as an effective supportive non-pharmacological intervention in oncology settings, particularly for lung cancer patients undergoing chemotherapy. VR’s capacity to decrease the perception of chemotherapy session duration could substantially improve patient comfort and treatment adherence. Moreover, the absence of adverse events related to VR use highlights its safety within the clinical setting. Healthcare professionals, including nursing staff, are encouraged to consider the integration of VR alongside other non-pharmacological interventions, as suggested by the literature [60], to enrich the support offered to cancer patients, potentially transforming the patient experience during challenging treatments. Participants appreciated the VR intervention, and its use reported no adverse side effects. Moreover, it is well known that the first cycle of chemotherapy is the least ‘disabling’. In this regard, we think that the study should be repeated in patients undergoing several sessions of chemotherapy, possibly even with cross-over groups, in order to thoroughly study the effects of this non-toxic methodology on a generally very disabling and impactful therapeutic intervention.

## 7. Conclusions

In conclusion, immersive VR represents a promising non-pharmacological strategy to reduce the chemotherapy discomfort and side effects for lung cancer patients. Providing a valuable and safe distraction that positively modifies the perception of time, VR has made chemotherapy sessions feel shorter and more tolerable to patients. While our study highlights the feasibility and safety of VR interventions in lung cancer care, further research is needed to elucidate its effects on treatment adherence and long-term patient outcomes. Investigating the effects of VR on a broader spectrum of symptoms and psychological outcomes and its cost-effectiveness could provide more comprehensive insights into its potential as a supportive tool in healthcare. Integrating VR into oncology care strategies offers a modern, patient-centered approach to alleviating the burdens associated with cancer treatment, emphasizing the need for continued innovation and evaluation in cancer care and nursing in clinical practices.

## Figures and Tables

**Table 1:** Socio-demographic characteristics of cancer patients (N = 100).

Socio-Demographic Variables	Virtual Reality Group (n = 55)	Control (n = 45)	Total (n = 100)	*p*-Value
Age (years)				0.058
Mean (SD)	67.4 (7.3)	67.2 (8.5)	67.3 (7.8)	
Median	66	68	67	
Minimum	53	49	49	
Maximum	81	81	81	
Civil Status n. (%)				1.517
Single	1 (1.8)	1 (2.2)	2 (2.0)	
Married	37 (67.3)	30 (66.7)	67 (67)	
Cohabitant	2 (3.6)	4 (8.9)	6 (6.0)	
Separated or divorced	8 (14.5)	6 (13.3)	14 (14.0)	
Widower	7 (12.7)	4 (8.9)	11 (11.0)	
Living with n. (%)				0.192
Alone	8 (14.5)	8 (17.8)	16 (16.0)	
With others	47 (85.5)	37 (82.2)	84 (84.0)	
Provenience n. (%)				0.278
Rome	29 (55.8)	23 (52.3)	52 (54.2)	
Same Region	14 (26.9)	14 (31.8)	28 (29.2)	
Outside Region	9 (17.3)	7 (15.9)	16 (16.7)	
Education n. (%)				4.314
Illiterate	1 (1.8)	0 (0.0)	1 (1.0)	
Primary School	8 (14.5)	7 (15.9)	15 (15.2)	
Secondary School	15 (27.3)	12 (27.3)	27 (27.3)	
High School	29 (52.7)	12 (43.2)	48 (48.5)	
Bachelor’s Degree	2 (3.6)	6 (13.6)	8 (8.1)	
Employment n. (%)				0.921
Unemployed	5 (9.3)	3 (6.8)	8 (8.2)	
Self-employed	5 (9.3)	3 (6.8)	8 (8.2)	
Employee	5 (9.3)	5 (11.4)	10 (10.2)	
Retired	37 (68.5)	30 (68.2)	67 (68.4)	
Other	2 (3.7)	3 (6.8)	5 (5.1)	
Posted by n. (%)				4.027
General Practitioner	4 (7.3)	0(0.0)	4 (4.3)	
Specialist	45 (81.8)	36 (94.7)	81 (87.1)	
Other	55 (10.9)	38 (5.3)	93 (8.6)	
Onset of pain n. (%)				0.087
No pain	36 (65.5)	30 (66.7)	66 (66.0)	
<3 months	8 (14.5)	7 (15.6)	15 (15.0)	
>3 months	11 (20.0)	8 (17.8)	19 (19.0)	
Type of pain n. (%)				1.678
Continuous pain	10 (18.2)	8 (17.8)	18 (18.0)	
Intermittent pain	6 (10.9)	7 (15.6)	13 (13.0)	
Other	4 (7.3)	1 (2.2)	5 (5.0)	

**Table 2:** Impact of immersive virtual reality on the Edmonton Symptom Assessment Scale.

Edmonton Symptom Assessment Scale Results											
Variables Pre-Chemotherapy	Groups	N	Mean	Std. Deviation	*p*-Value	Variables Post-Chemotherapy	Groups	N	Mean	Std. Deviation	*p*-Value
Pain (NRS)	VR	55	1.60	2.705	0.69	Pain (NRS)	VR	53	1.11	1.948	0.26
	control	45	1.82	2.847			control	45	1.67	2.900	
Tiredness (NRS)	VR	55	3.73	2.990	0.73	Tiredness (NRS)	VR	53	2.83	3.155	0.71
	control	45	3.51	3.210			control	45	3.07	3.179	
Nausea (NRS)	VR	55	0.93	2.053	0.42	Nausea (NRS)	VR	53	0.49	1.339	0.39
	control	45	0.62	1.585			control	45	0.76	1.694	
Depression (NRS)	VR	55	2.51	3.231	0.86	Depression (NRS)	VR	53	1.49	2.383	0.12
	control	45	2.40	3.018			control	45	2.36	3.098	
Anxiety (NRS)	VR	55	2.93	3.399	0.82	Anxiety (NRS)	VR	53	2.68	3.221	0.84
	control	45	2.78	2.899			control	45	2.56	2.865	
Drowsiness (NRS)	VR	55	1.93	2.441	0.24	Drowsiness (NRS)	VR	53	1.75	2.638	0.49
	control	45	2.56	2.833			control	45	2.11	2.525	
Appetite (NRS)	VR	55	1.60	2.671	0.31	Appetite (NRS)	VR	53	1.04	1.839	0.08
	control	45	2.18	3.040			control	45	1.87	2.793	
Feeling of well-being (NRS)	VR	55	2.64	3.081	0.95	Feeling of well-being (NRS)	VR	53	1.51	2.628	0.23
	control	45	2.60	2.957			control	45	2.18	2.847	
Shortness of breath (NRS)	VR	55	2.31	2.860	0.40	Shortness of breath (NRS)	VR	53	2.45	3.226	0.73
	control	45	2.82	3.249			control	45	2.69	3.377	

Legend: ESAS = Edmonton Symptom Assessment Scale; NRS = Numeric Rating Scale (0–10); VR = virtual reality.

**Table 3:** Results of vital parameters using virtual reality.

Vital Parameter Results											
Variables Pre-Chemotherapy	Groups	N	Mean	Std. Deviation	*p*-Value	Variables Post Chemotherapy	Groups	N	Mean	Std. Deviation	*p*-Value
Systolic blood pressure (mmHg)	VR	54	124.57	21.744	0.67	Systolic blood pressure (mmHg)	VR	53	126.04	12.340	0.88
	control	45	123.02	12.636			control	45	125.67	12.995	
Diastolic blood pressure (mmHg)	VR	54	72.69	7.753	0.07	Diastolic blood pressure (mmHg)	VR	53	73.49	7.311	0.27
	control	45	69.76	8.060			control	45	71.73	8.256	
Cardiac frequency (n/min)	VR	54	78.52	11.118	0.64	Cardiac frequency (n/min)	VR	53	77.43	10.300	0.68
	control	45	79.64	12.851			control	45	76.51	11.555	
Respiratory frequency (n/min)	VR	54	13.22	2.567	0.86	Respiratory frequency (n/min)	VR	52	13.04	2.275	0.20
	control	44	13.32	2.963			control	44	13.77	3.333	
Body temperature (°Celsius)	VR	54	36.143	.2124	0.66	Body temperature (°Celsius)	VR	53	36.108	.2716	0.87
	control	45	36.162	.2229			control	45	36.116	.1930	
Oxygen saturation (%)	VR	54	97.00	1.427	0.02	Oxygen saturation (%)	VR	53	97.19	1.241	0.08
	control	45	96.22	1.820			control	45	96.64	1.786	

Legend: mmHg = millimeters of mercury.

## Data Availability

Data that support the findings herein reported will be made available through material transfer agreement upon reasonable request.

## Supplementary Materials

The following supporting information can be downloaded at: https://www.mdpi.com/article/10.3390/jcm13154374/s1, Table S1: Transparent Reporting of Evaluations with Nonrandomized Designs (TREND) Statement Checklist; Table S2: Questionnaire on socio-demographic information; Table S3: Post-Intervention questionnaire for intervention group; Table S4: Post-Intervention questionnaire for comparison group; Table S5: Checklist for data collection by nursing staff.
